# In-Depth Endogenous Phosphopeptidomics of Serum with Zirconium(IV)-Grafted Mesoporous Silica Enrichment

**DOI:** 10.1021/acs.analchem.3c02150

**Published:** 2024-05-10

**Authors:** Ci Wu, Shen Zhang, Chunyan Hou, Stephen Byers, Junfeng Ma

**Affiliations:** † Department of Oncology, Lombardi Comprehensive Cancer Center, Georgetown University Medical Center, Washington D.C. 20007, United States; ‡ Clinical Research Center for Reproduction and Genetics in Hunan Province, Reproductive and Genetic Hospital of CITIC-XIANGYA, Changsha 410000, China; ⊗ School of Chemistry and Chemical Engineering, Liaoning Normal University, Dalian 116029, China

## Abstract

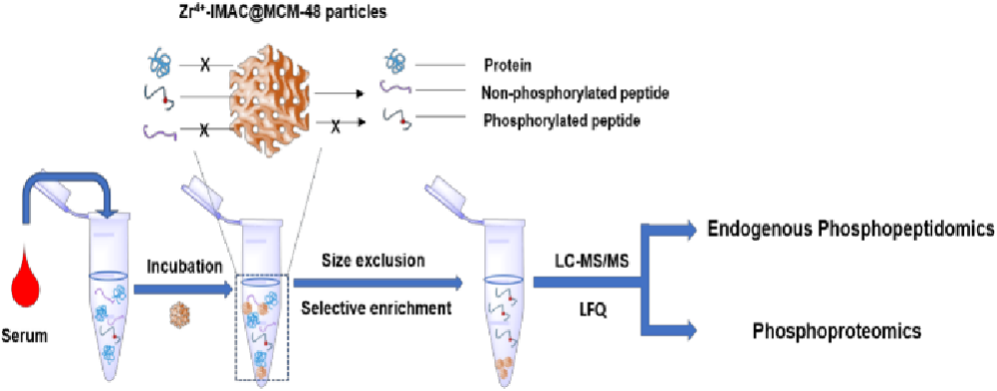

Detection of endogenous peptides, especially those with modifications (such as phosphorylation) in biofluids, can serve as an indicator of intracellular pathophysiology. Although great progress has been made in phosphoproteomics in recent years, endogenous phosphopeptidomics has largely lagged behind. One main hurdle in endogenous phosphopeptidomics analysis is the coexistence of proteins and highly abundant nonmodified peptides in complex matrices. In this study, we developed an approach using zirconium(IV)-grafted mesoporous beads to enrich phosphopeptides, followed by analysis with a high resolution nanoRPLC-MS/MS system. The bifunctional material was first tested with digests of standard phosphoproteins and HeLa cell lysates, with excellent enrichment performance achieved. Given the size exclusion nature, the beads were directly applied for endogenous phosphopeptidomic analysis of serum samples from pancreatic ductal adenocarcinoma (PDAC) patients and controls. In total, 329 endogenous phosphopeptides (containing 113 high confidence sites) were identified across samples, by far the largest endogenous phosphopeptide data set cataloged to date. In addition, the method was readily applied for phosphoproteomics of the same set of samples, with 172 phosphopeptides identified and significant changes in dozens of phosphopeptides observed. Given the simplicity and robustness of the proposed method, we envision that it can be readily used for comprehensive phosphorylation studies of serum and other biofluid samples.

## Introduction

Endogenous peptides, the natural form of polypeptides of 3–100 amino acids, are widely distributed in tissues, cells, and body fluids.^1,2^ Endogenous peptides are mainly generated through the cleavage of precursor proteins (e.g., by proteases/peptidases),^3^ gene-independent enzymatic formation (from amino acids), and gene-encoding (bioactive peptides).^4,5^ Due to their importance in health and disease, the analysis of endogenous peptides has drawn much attention.^6−9^ Of note, endogenous peptides undergo post-translational modifications such as phosphorylation. Endogenous phosphopeptides in biofluids (e.g., serum), either degraded from larger proteins or secreted from cells and tissues, serve as a critical indicator for physiopathologic events. Emerging evidence suggests that characterization of endogenous phosphopeptides in human serum can provide valuable insights into the physiological and pathological status of an individual, as well as potential biomarkers for disease diagnosis and monitoring.^10,11^

In contrast to phosphoproteomics, comprehensive characterization of endogenous phosphopeptides existing in biofluids (e.g., serum) has been technically challenging. Despite the presence of atypical Fam20 secretory pathway kinases,^12^ phosphorylation levels on proteins appear to be extremely low in sera (largely due to the active phosphatases).^13^ Detection of endogenous phosphopeptides is further exacerbated by the complex matrices in which they are present. To enable identification of endogenous peptides, high abundant proteins in serum often have to be depleted beforehand (e.g., by organic-solvent-assisted protein precipitation, filter-mediated ultracentrifugation, and mesoporous beads).^14−16^ To achieve sensitive analysis of endogenous phosphopeptides with even lower abundance, robust and highly selective enrichment is indispensable. Of note, despite the great progress made toward deep phosphoproteomics in recent years (which is based mainly on metal oxide affinity chromatography (e.g., TiO_2_ beads) and immobilized metal ion affinity chromatography (e.g., Zr^4+^/Ti^4+^-IMAC)), methods for phosphoproteomics are not inherently adaptable to endogenous phosphopeptidomics.^17−19^ To maximize enrichment of endogenous phosphopeptides, multiple sample preprocessing strategies have been exploited, including organic solvent precipitation and lipid removal from the matrices.^20^ As a promising alternative, porous materials (especially mesoporous materials) bearing a phosphate-specific affinity have gained popularity due to their unique properties to exclude proteins (as well as other large molecules) and thus direct enrichment of endogenous phosphopeptides. For example, porous metal–organic framework materials (MOFs) were prepared and modified with metal oxide (e.g., Fe_3_O_4_) or metal ions (e.g., Ti^4+^) for the analysis of endogenous phosphopeptides.^21−24^ In addition, several different types of mesoporous materials were exploited to enrich endogenous phosphopeptides.^25−27^ Although these materials showed excellent performance for enriching the four high-abundance endogenous phosphopeptides in serum samples, their capacity for other phosphopeptides has not been investigated. Recently, Zou et al. tentatively explored endogenous phosphopeptidomics by integrating selective enrichment of phosphopeptides, offline HPLC fractionation (into 9 fractions), and nanoflow RPLC-MS/MS system with complementary fragmentation techniques.^28^ With this approach, 143 endogenous phosphopeptides were identified from 500 μL of the control serum samples. Despite the success, clearly there is a strong need to develop simplified and robust yet efficient methods for the deep analysis of endogenous phosphopeptidomics.

To that end, we established a new strategy for the analysis of endogenous phosphopeptides in serum samples. Specifically, mesoporous silica MCM-48 beads (with a pore diameter of ∼2.5 nm) were utilized for the immobilization of Zr^4+^ in a straightforward and robust manner. After being successfully tested with digests of standard proteins and HeLa cell lysates, MCM-48-based Zr^4+^-IMAC was applied for direct analysis of the endogenous phosphopeptidome of serum samples from pancreatic ductal adenocarcinoma (PDAC) and control individuals. With this substantially simplified one-pot enrichment procedure, 329 endogenous phosphopeptides were identified and quantified, benchmarking deep analysis of endogenous phosphopeptides. Last but not least, the method also allowed simultaneous phosphoproteomics for the same set of serum samples.

## Experimental Section

### Sample Preparation

Tryptic digests of α-casein, β-casein, and BSA were prepared by digesting with trypsin at a ratio of 1/50 (enzyme/substrate, w/w) at 37 °C overnight. The digests were desalted with a C18 spin column and dried in a SpeedVac (Fisher Scientific).The digests of HeLa cell lysates and serum were prepared by using S-Trap columns with a procedure described previously.^29^ 500 μL of the human serum sample spiked with a panel of inhibitors (consisting of 2 μM pepstatin A, 10 μM bestatin hydrochloride, and 1 μM marimastat) was diluted with 5 mL of the loading buffer (containing 80% ACN, 5% TFA, and 0.1 M glycolic acid). The solution was then centrifuged at 12 000 g for 10 min at 4 °C, with the supernatant collected for further processing. Details can be seen from the “Experimental Section” in the Supporting Information.

### Enrichment of Trypsin Digestion-Derived Phosphopeptides and Endogenous Phosphopeptides

**Figure 1:**
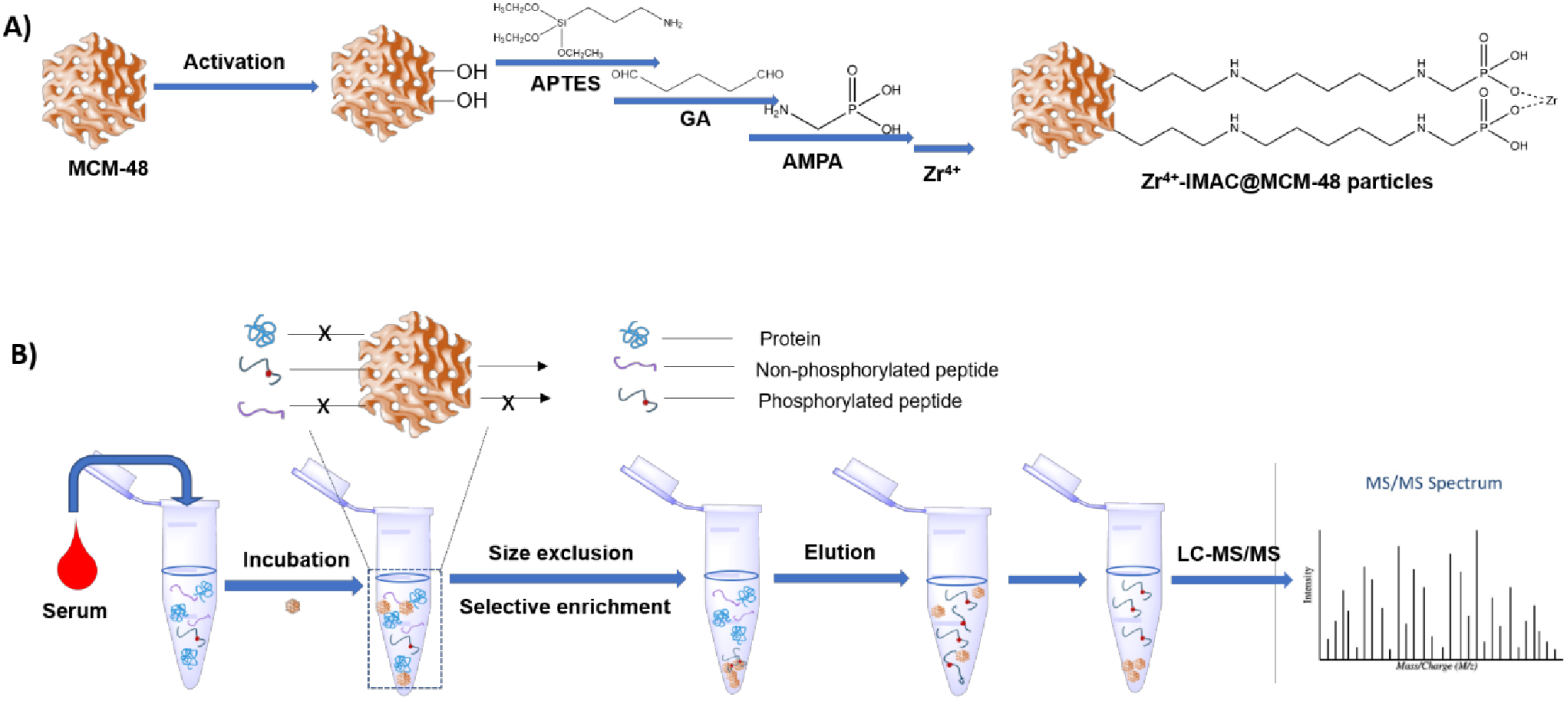
(A) Scheme for the preparation of Zr-IMAC@MCM-48 particles. (B) Procedure for the analysis of the phosphopeptides.

The workflow for the analysis of endogenous phosphopeptides from human serum samples is shown in Figure 1B. In brief, the endogenous phosphopeptides from serum were enriched by incubating the serum sample with Zr-IMAC@MCM-48 particles (2 mg). 500 μL amount of serum was suspended in the loading buffer (80% ACN, 5% TFA, 0.1 M glycolic acid) and centrifuged for 15 min at 15 000 rpm. Then, the supernatant was incubated with Zr-IMAC@MCM-48 beads for 1 h at room temperature. After centrifugation at 5000 rpm for 2 min, the supernatant was removed and the beads were washed with 400 μL of 80% acetonitrile, 1% TFA, and then 400 μL of 0.1% TFA. Finally, the peptides were eluted with 50 μL of 1% ammonium hydroxide and 50 μL of 5% pyrrolidine sequentially. The combined eluates were desalted with a C18 micro spin column and dried in a SpeedVac. (Please see the “Experimental Section” in Supporting Information for more details).

## Results and Discussion

### Synthesis and Characterization of Zr-IMAC@MCM-48

Simplified and specific enrichment of endogenous phosphopeptides is critical for their analysis in serum and other biofluids. In the present study, porous silica MCM-48 beads were exploited given their highly uniform mesopore size, which is inherently beneficial for size exclusion of large size molecules (e.g., proteins). To achieve specific capture of phosphopeptides, Zr^4+^ was grafted onto the mesoporous beads via a facile manner (Figure 1A). Briefly, MCM-48 microspheres were first treated with acids to produce enough −OH groups for modification and then sequentially activated with APTES and glutaraldehyde. A heterofunctional reagent AMPA was then introduced to yield functional −PO_3_^2–^ groups, enabling Zr^4+^ immobilization onto the homogeneous mesopores of MCM-48 via chelation.

**Figure 2:**
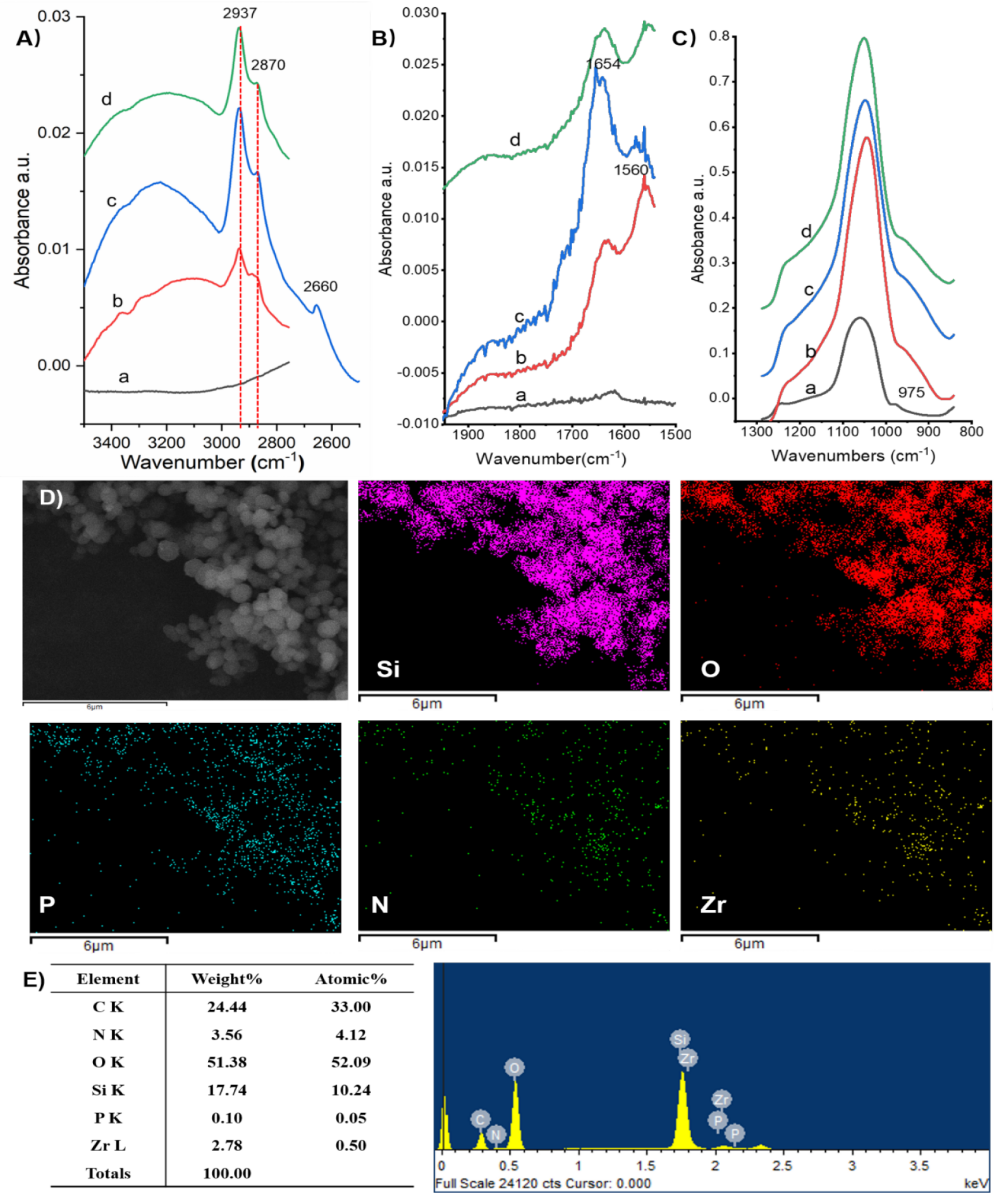
Three segmented ATR spectra: (A) 3500–2500 cm^–1^, (B) 1950–1500 cm^–1^, and (C) 1350–800 cm^–1^ of the MCM-48 beads: (a) original MCM-48 beads, (b) APTES modified MCM-48 beads, (c) glutaraldehyde modified MCM-48 beads, and (d) AMPA modified MCM-48 beads. (D) Elemental mapping images of Zr-IMAC@MCM-48 beads. (E) EDX spectrum and chemical composition (table) of Zr-IMAC@MCM-48 beads.

FTIR spectra were taken to confirm each of the reaction steps for the preparation of MCM-48-based Zr-IMAC beads, with the entire spectrum of the materials shown in Figure S1A. The characteristic absorption peaks of the functional groups were displayed in three segmented ATR spectra of 3500–2500, 1950–1500, and 1350–800 cm^–1^. The adsorption bands at 2870 cm^–1^ (stretching) and 2937 cm^–1^ (bending) attributing to C–H vibrations showed up after APTES modification (Figure 2A), suggesting that the amino groups were effectively grafted on the mesoporous silica.^30,31^ The strong bands at 1654 and 2660 cm^–1^, which could be assigned to −CHO vibrations, appeared after glutaraldehyde treatment (Figure 2B), suggesting that aldehyde groups were anchored onto the MCM-48 material. The disappearance of the peak of 1654 cm^–1^, together with the negligible peak at 975 cm^–1^ (corresponding to Si–OH; Figure 2C) after further treatment with AMPA, demonstrates the successful modification of functional −PO_3_^2–^ groups onto the mesopores of MCM-18 beads.^32^ EDX spectroscopic analysis revealed that Zr-IMAC@MCM-48 materials are composed of silica, carbon, oxygen, nitrogen, phosphorus, and zirconium (Figure 2D). It is estimated that the weight and atom percentages of zirconium are 2.8% and 0.5% (Figure 2E), respectively. Furthermore, the particle size distribution (i.e., cumulative distribution and differential distribution) measurement (Figure S1B) suggests that the Zr-IMAC@MCM-48 material is well-distributed. Surface moment mean diameter (or Sauter mean diameter) is measured to be 14.9 μm, and the volume moment mean diameter (or De Brouckere mean diameter) is 36.2 μm. In addition, nitrogen adsorption–desorption isotherm analysis reveals that the beads exhibit an average pore diameter of 2.5 nm and a Brunauer–Emmett–Teller (BET) specific surface area of 141.1 m^2^/g. Therefore, the Zr-IMAC@MCM-48 beads have combined properties of ordered mesopores (2.5 nm) and Zr^4+^-based specific chelation toward phosphate groups, enabling direct enrichment of endogenous phosphopeptides from serum samples.

### Performance Evaluation of Zr-IMAC@MCM-48 Beads

To ensure its successful application for endogenous phosphopeptides, we tested its enrichment performance by using digests of standard phosphoproteins and HeLa cells, given the unavailability of standards for endogenous phosphopeptides. The selectivity of Zr-IMAC@MCM-48 for phosphopeptide capture was initially evaluated by using tryptic digests of the standard phosphoprotein β-casein by MALDI-TOF analysis. As shown in Figure S2A, for the digest without enrichment, the mass spectrum was dominated by signals of unmodified peptides, with only one phosphorylated peptide detected. However, clear signals for all three phosphopeptides (at *m*/*z* 2061.9, *m*/*z* 2556.2, and *m*/*z* 3122.2) were observed (without interference from nonphosphorylated peptides) for the sample after enrichment (Figure S2B). Furthermore, when BSA and β-casein tryptic digests were mixed at a mass ratio of 1000:1, phosphopeptides were undetectable due to suppression by a high abundance of unmodified peptides (Figure S2C). In contrast, the three phosphopeptides were distinctly detected (with minimal interference from other peptides) after enrichment (Figure S2D). More remarkably, for the digests mixing at an even higher interfering mass ratio of 5000:1, the phosphopeptide at *m*/*z* 2061.9 was still detectable (Figure S2E,F), highlighting its exceptional enrichment specificity.

The enrichment performance of the Zr-IMAC@MCM-48 material was further evaluated by using the tryptic digest of HeLa cell lysates. Numerous IMAC materials (coordinated with various transition metal ions such as Ti^4+^, Fe^3+^, and Zr^4+^)^33−39^ and MOAC materials (such as ZrO_2_ and TiO_2_)^40−43^ have been reported to enrich complementary phosphopeptides. Herein, to systematically evaluate the performance of our material, a head-to-head and rigorous comparison of the performance of Zr-IMAC@MCM-48, Ti-IMAC@MCM-48, and Fe-IMAC@MCM-48 was conducted by using the same approach. The performance of these materials, along with that of the commercial Fe-NTA and TiO_2_ beads, was additionally assessed by analyzing the enriched phosphopeptides. The results of phosphopeptides, phosphoproteins, and phosphorylated sites of all the other materials were shown in Figure S3A. The numbers of identified phosphoproteins, phosphopeptides and phosphosites by the four materials were: Zr-IMAC@MCM-48 (2411, 8314, and 7076), Ti-IMAC@MCM-48 (2463, 8366, and 6558), Fe-NTA (2131, 7075, and 6294) and TiO_2_ (2676, 8484, and 6513), respectively. Unfortunately, no phosphopeptides were captured by Fe-IMAC@MCM-48, likely due to the low valence state (+3) of iron ions and thus insufficient complexation with both the backbone of the AMPA-based material and the phosphate group on peptides. Overall, our Zr-IMAC@MCM-48 beads display an enrichment performance comparable to that of the classical materials (i.e., Fe-NTA and TiO_2_). Moreover, our analysis results are comparable to or even better than a number of recent phosphoproteomics studies in which equal or similar amounts of HeLa cell digests were used, and single-shot mass spec. analyses were performed.^27,33^,^37^,^44−52^ Given that phosphorylation levels in serum samples are much lower (in contrast to complex samples such as whole cell lysates and tissue samples), we think it is decent enough to perform single-shot analyses and comparisons (rather than doing extensive fractionation for DDA-based phosphoproteomics or DIA-based phosphoproteomics).^53−57^ While many phosphosites were identified by different approaches, still a distinct set of phosphosites could be found by each method (Figure S3B). As shown in Figure S3C, a total of 8314 phosphopeptides were identified after Zr-IMAC@MCM-48 enrichment, comprising 5680 singly (68.4%), 2058 doubly (24.8%), and 572 triply phosphorylated peptides (6.9%), which is similar to the results obtained with Fe-NTA. In contrast, TiO_2_ and Ti-IMAC enrichment methods yielded a higher percentage of singly phosphopeptides (88.2% and 81.2%, respectively). In terms of phosphosite identification, as illustrated in Figure S3D, all beads display similar enrichment toward phosphoserine, phosphothreonine, and phosphotyrosine. The physicochemical properties of the phosphopeptides obtained through various materials, including the distribution of pI and GRAVY values, are illustrated in Figure S3E,F. The findings indicate that Zr-IMAC and Fe-NTA demonstrate a more substantial advantage in the identification of acidic phosphopeptides with pI < 4 compared to TiO_2_ and Ti-IMAC. Additionally, the GRAVY distributions of these four materials do not show significant differences. The enrichment selectivity (the percentage of phosphopeptides in total peptides identified) reached as high as 78.2%. Overall, our Zr^4+^-IMAC@MCM-48 beads display comparable enrichment performance to those of other classical materials, and different materials exhibit a distinct affinity for purifying diverse species of phosphopeptides.

Finally, three replicates were conducted for the enrichment of phosphopeptides from the tryptic digest of HeLa cell lysates to evaluate the reproducibility of Zr-IMAC@MCM-48. The numbers of phosphopeptides identified were 7941, 7667, and 7959 (with a RSD of 2.1% (*n* = 3)) (Figure S6A). Of note, almost 70% of the phosphopeptides were identified in at least two runs (Figure S6B), suggesting great reproducibility for phosphopeptide enrichment.

### Endogenous Phosphopeptidomics Using Zr-IMAC@MCM-48

Although great enrichment performance of Zr-IMAC@MCM-48 for phosphoproteomics is obtained, the ultimate goal of this study is to develop a method for endogenous phosphopeptidomics (e.g., in serum). Different from digests from complex samples (e.g., HeLa cell lysates aforementioned), human serum contains not only endogenous peptides but also many other molecules (e.g., abundant proteins).^39,57,58^ Although IMAC/MOAC-based enrichment alone has been attempted for endogenous phosphopeptidomics, only limited success has been achieved,^14,20^ largely due to serum complexity and the ion suppression effect by proteins/nonphosphopeptides. To validate the protein removal performance, SDS-PAGE was employed to analyze the protein composition before and after the enrichment of endogenous phosphopeptides in serum. For direct comparison, samples processed with Fe-NTA and TiO_2_ materials were also analyzed by SDS-PAGE (Figure S4A). Compared to the nonenriched samples, no protein bands were visualized for samples enriched by Zr-IMAC@MCM-48, indicating a high efficiency of protein removal. However, some protein bands remained visible after treatment with Fe-NTA and TiO_2_ materials (Figure S4A). Moreover, the samples enriched with Zr-IMAC@MCM-48 beads did not give protein peaks in downstream mass spectrometry analysis. By analyzing the quality control (QC) samples after running multiple endogenous phosphopeptide samples enriched from serum (Figure S4B), we found a high protein overlap across three QC runs, demonstrating its excellent capability to eliminate interference proteins. Furthermore, to evaluate the enrichment efficacy of Zr-IMAC@MCM-48 beads, digests of α-casein and β-casein, both with and without spiked into serum, were analyzed. A total of 30 phosphopeptides (including 27 phosphopeptides from α-casein and 3 from β-casein, Table S1) were identified from the digests. Among the phosphopeptides identified, many were matched with two or more phosphorylation sites, indicating that both singly phosphorylated peptides and multiply phosphorylated peptides can be well enriched without bias. Moreover, 24 phosphopeptides were successfully detected from the digests of α-casein and β-casein spiked into serum, indicating great promise for endogenous phosphopeptide analysis.

To avoid potential proteolytic cleavage of proteins during sample processing, a cocktail of inhibitors (including pepstatin A, bestatin, and marimastat) were spiked into each sample before analysis. Zr-IMAC@MCM-48 beads were used for direct capture of endogenous phosphopeptides of serum samples from five PDAC and five healthy control participants. In total, 329 endogenous phosphopeptides (containing 113 high confidence sites) from 64 precursor proteins were identified across samples. Our study provides the largest data set of endogenous phosphopeptides in serum samples to date, in comparison to previous studies (most of which only identified very limited number of endogenous phosphopeptides (i.e., the most abundant ones)) (as shown in Table S2). Of note, only 2 phosphopeptides (with 1 confident phosphorylated site) were identified by using particles without immobilized metal ions, demonstrating that Zr^4+^-chelation is key to enable enrichment selectivity. The detailed information on identified endogenous phosphopeptides and phosphosites in this study is shown in Table S3. Moreover, an enrichment specificity of 61% was achieved, much higher than other endogenous phosphopeptide strategies (∼20% on average).^20^ The unique properties of the Zr-IMAC@MCM-48 beads (e.g., mesopores-based size-exclusion and Zr-IMAC-based specific enrichment), together with the substantially simplified workflow, are key contributors to the deep endogenous phosphopeptidomics in serum.

**Figure 3:**
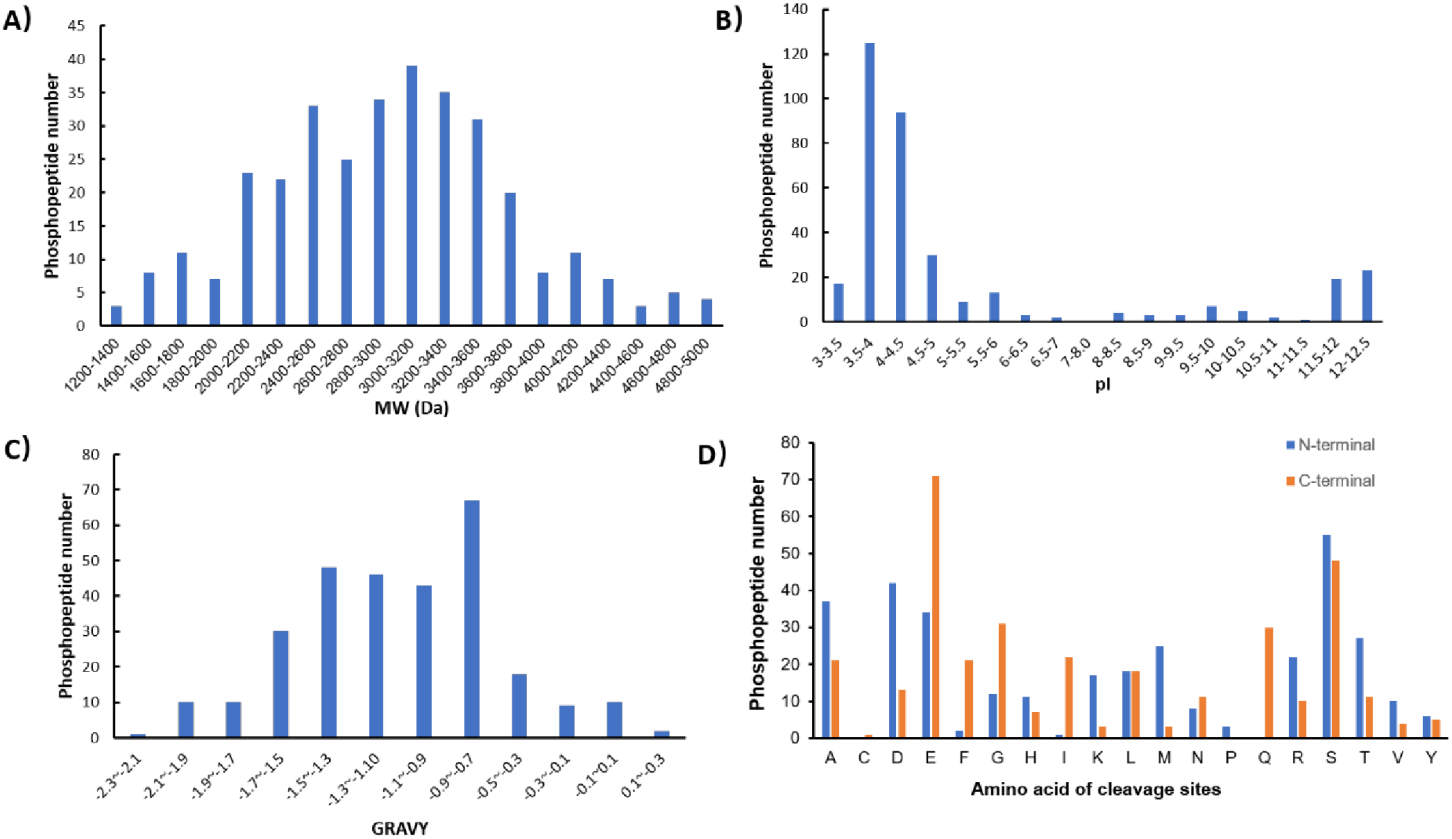
Physiochemical properties of serum endogenous phosphopeptides enriched by Zr-IMAC@MCM-48. (A) Distribution of the molecular weight (MW). (B) Distribution of the isoelectric point (pI). (C) Distribution of GRAVY values. (D) Amino acids at the N-terminus and C-terminus.

Moreover, the physiochemical properties of those identified endogenous phosphopeptides were systematically investigated. The MW of the identified phosphopeptides was in the range of 1200–5000 (Figure 3A). Given its highly uniform pore size of 2.5 nm, it is within anticipation that large *m*/*z* endogenous phosphopeptides up to 5000 Da can be captured using the bifunctional material. With the further increase of the pore size, endogenous phosphopeptides with an even higher MW may also be identified. Regarding the distribution of the isoelectric point (pI), phosphopeptides spread over a broad range of pI from 3 to 12.5 (Figure 3B). It appears that most of them are acidic peptides (mainly in the range of 3–5), in agreement with previous reports.^20^ The hydrophobicity of the identified peptides was also investigated, as illustrated by the grand average hydrophobicity (GRAVY) values (Figure 3C). It turned out that the GRAVY values of phosphopeptides were in the range of −2.3 to 0.3 with an average value of −1.00, indicating that most of the endogenous phosphopeptides identified in this work were hydrophilic. The residues at the N-terminal and C-terminal of the identified phosphopeptides have been considered as clues on the action mechanisms of proteases which might have produced cleavage sites of the identified peptides from precursor proteins. As shown in Figure 3D, the most frequently observed amino acids at the C-terminus are glutamic acid (E), serine (S), glycine (G), and glutamine (Q), while N-terminal were serine (S), aspartic acid (D), alanine (A), and glutamic acid (E). Furthermore, it was found that among the 113 highly confident phosphorylation sites, 82% (93), 15% (17), and 3% (3) are phosphoserine, phosphothreonine, and phosphotyrosine, respectively. In addition, the percentages of singly and multiply phosphorylated peptides were calculated to be 67% (219) and 33% (110), respectively, demonstrating excellent enrichment performance for multiply phosphorylated peptides. The representative MS^2^ spectra of endogenous phosphopeptides including 1 site, 2 sites, and 3 sites are shown in Figure S5. Last but not least, we performed three enrichment replicates for endogenous phosphopeptides from serum, with an identification RSD of 4.5% achieved (*n* = 3) and 63.5% of phosphopeptides identified in at least two runs (Figure S6A,C), indicating good analytical reproducibility for endogenous phosphopeptides. The dynamic range of the identified endogenous phosphopeptides was found to span roughly over 5 orders of magnitude (Figure S7).

### Comparative Analysis of the Serum Endogenous Phosphopeptidome and Phosphoproteome from PDAC Patients and Healthy Individuals

Pancreatic ductal adenocarcinoma (PDAC) is one of the most aggressive tumors, with a median survival of six months, largely due to the late diagnosis, early metastasis, and limited response to therapeutics. Since a large proportion of patients are diagnosed at an advanced stage, there are often very scarce therapy options.^59,60^ The identification of biomarkers for the early diagnosis of pancreatic cancer is of great importance. Hence, comprehensive analysis of serum phospho-proteins/peptides from PDAC holds promise for the discovery of novel diagnostic biomarkers.^61^

**Figure 4:**
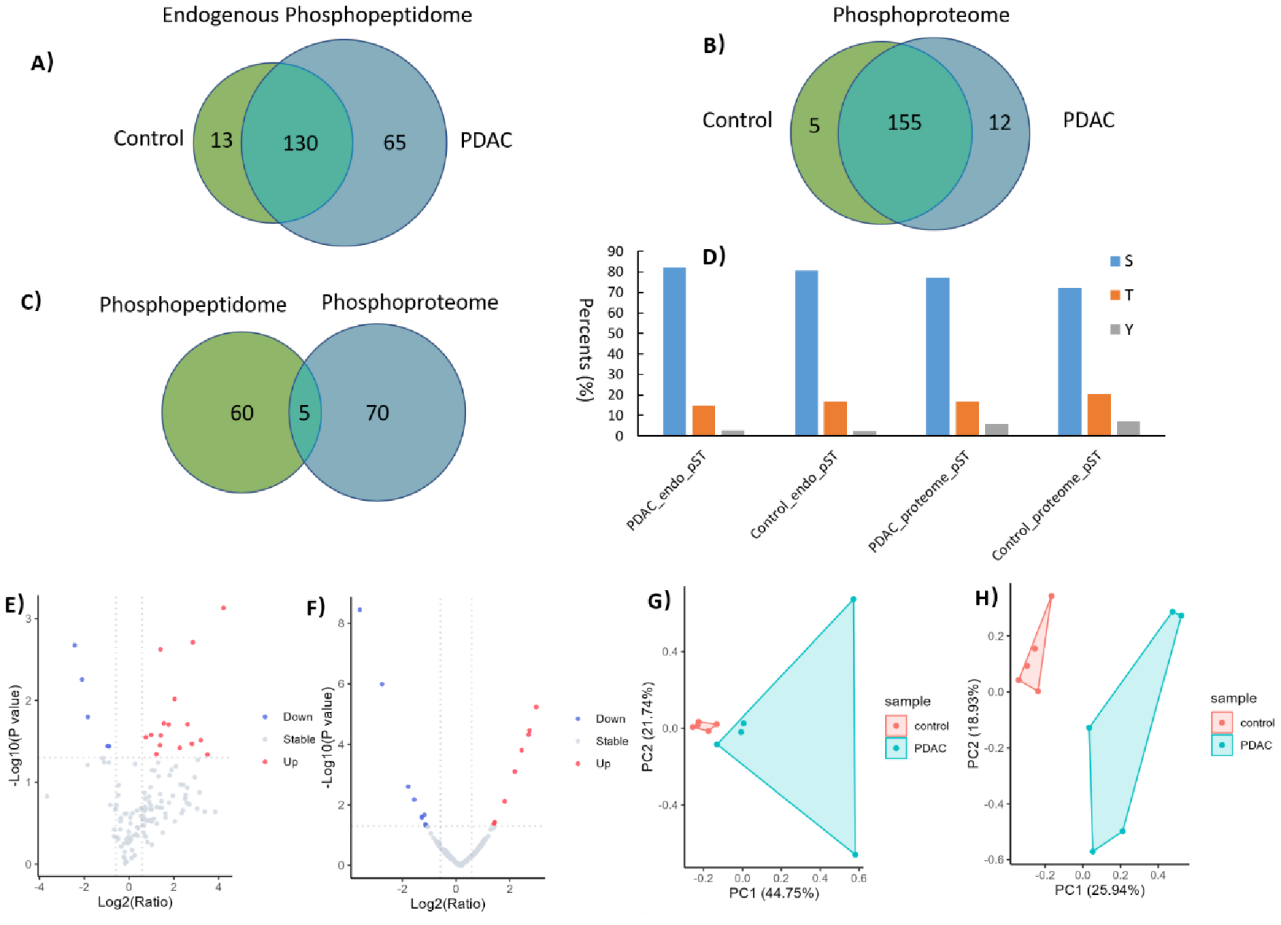
Characterization of serum endogenous and trypsin digestion-derived phosphopeptides enriched by Zr-IMAC@MCM-48. (A) Overlap of endogenous phosphopeptides identified from PDAC and control serum. (B) Overlap of phosphopeptides resulted from phosphoproteomics of PDAC and control serum samples. (C) Overlap of the precursor phosphoproteins identified from phosphopeptidome and phosphoproteome analyses. (D) Distribution of phosphorylation on serine (S), threonine (T), and tyrosine (Y) for endogenous phosphopeptides. Volcano plots of (E) endogenous phosphopeptides and (F) trypsin digestion-derived phosphopeptides. Principle component analysis of (G) endogenous phosphopeptides and (H) trypsin digestion-derived phosphopeptides.

Since our material Zr-IMAC@MCM-48 showed excellent enrichment of both endogenous phosphopeptides and trypsin digestion-derived phosphopeptides, we performed comprehensive phosphorylation analysis at both endogenous phosphopeptidome and phosphoproteome levels. As shown in Figure 4A, in total, 208 endogenous phosphopeptides (from 65 precursor phosphoproteins) were quantified. Among them, 65 and 13 phosphopeptides were uniquely identified in PDAC and control serum (Table S4), respectively. While 130 endogenous phosphopeptides were present in both groups (Table S4). Regarding phosphoproteomics, tryptic digestion followed by Zr-IMAC@MCM-48 enrichment yielded 172 phosphopeptides (corresponding to 75 proteins; Figure 4B). Among them, 11 and 5 phosphopeptides were uniquely identified in PDAC and control serum, respectively (Table S5). Of the phosphosites identified, slightly higher percentages of phosphotyrosine (∼6%) were identified in the phosphoproteome compared to that of the endogenous phosphopeptidome (∼2%) (Figure 4D).

Interestingly, comparison between 65 precursor phosphoproteins from the endogenous phosphopeptidomics data set and 75 phosphoproteins from the phosphoproteomics data set shows that only 5 proteins are overlapped between the two studies (Figure 4C). Collectively, these results suggest that the endogenous phosphopeptidome and phosphoproteome are almost exclusively distinct populations. As each provides unique information, simultaneous analysis of both will be beneficial for obtaining a comprehensive map of phosphorylation events of serum samples.

Further quantitative analysis has enabled us to comprehensively evaluate a multitude of serum phosphorylation events in serum associated with PDAC. Among the 130 commonly identified endogenous phosphopeptides, 21 were significantly regulated in cancer compared to noncancer (Table S4, Figure 4E). Although eight endogenous phosphopeptides derived from fibrinogen alpha chain (P02671, FGA) were well quantified in our samples, none of them showed significant changes, including the four most abundant phosphopeptides (i.e., A.DpSGEGDFLAEGGGVR.G, A.DpSGEGDFLAEGGGV.R, T.ADpSGEGDFLAEGGGVR.G, and T.ADpSGEGDFLAEGGGV.R). Notably, seven endogenous phosphopeptides derived from Caveolae-associated protein 2 (O95810, Cavin2) were upregulated in PDAC. Interestingly, two phospopeptides (R.HPPVLpTPPDQEVIRN.I, R.GLpSWDSGPEEPGPRLQ.K) derived from protein kinase C (P05771, PKCB) were also upregulated in PDAC. Since Cavin2 is a proven substrate of PKCB, whether PCKB contributes to increased levels of phosphopeptides from Cavin2 in sera is worthy to be investigated. In addition, levels of two phosphopeptides corresponding to FOXK2 (a FOX family member which plays a critical role in cancer^62^ were upregulated in PDAC. Besides, increased phosphorylation has been observed in many other endogenous phosphopeptides (except the peptide MApSESDTEEFYDAPEDVH.L derived from WD repeat-containing protein 44 (Rabphilin-11)).

Quantitative phosphoproteomics analysis shows that 18 phosphopeptides are significantly changed in serum samples from the control and PDAC (Table S5; Figure 4F). Remarkably, increased phosphorylation on site Ser-2475 of fibronectin was observed in PDAC serum. Higher phosphorylation on two sites Ser-275 and Ser-280 of peptide (R.EFHpSHEFHpSHEDMLVVDPK.S) from protein SPP1 (osteopontin, P10451) was revealed. As osteopontin itself was proposed as a marker for pancreatic cancer,^63^ it is interesting to know whether its phosphorylation would provide additional discriminating power for PDAC.

Finally, principal component analysis (PCA) was performed for the endogenous phosphopeptidomics data set and the phosphoproteomics data set. The first component (PC1) and the second component (PC2) could clearly differentiate the PDAC group from control individuals using either endogenous phosphopeptides or trypsin digestion-derived phosphopeptides. The distinct serum phosphorylation profiles indicate that the obtained significantly changed endogenous/tryptic phosphopeptides (from Cavin2 or osteopontin) may serve as potential markers for PDAC. Of note, phosphopeptides in the healthy control group are tightly clustered, while a scattered plot is observed in the PDAC group (Figure 4G,H). This may be ascribed to the negligible differences in healthy individuals but relatively large disparities among PDAC patients.

Phosphorylation levels on endogenous peptides and proteins in blood are generally low, making the development of phosphoproteins as disease biomarkers from such biofluids a challenging task. To the best of our knowledge, this study represents the first quantitative serum endogenous phosphopeptidomics analysis for pancreatic cancer. Moreover, simultaneous analysis of the serum phosphoproteome is also beneficial for obtaining a comprehensive map of phosphorylation events in serum. Although further validation studies with large sample cohorts are needed, our results strongly suggest that monitoring the phosphorylation of proteins and endogenous peptides may provide discriminative power for the detection of PDAC.

## Conclusions

A novel material Zr-IMAC@MCM-48 was developed for the analysis of endogenous phosphopeptides in serum samples. The material demonstrated unique properties, including mesopore-based size-exclusion (which removes proteins and other macromolecules) and one-pot enrichment of phosphopeptides, enabling substantially simplified endogenous phosphopeptidomic analysis. The performance was benchmarked by quantitative endogenous phosphopeptidomics for serum samples from PDAC and healthy controls. Considering that Zr-IMAC@MCM-48 could also be used for phosphoproteomics analysis, it renders simultaneous and comprehensive characterization of phosphorylation events (i.e., phosphopeptidomics and phosphoproteomics) in a given set of samples. Facile and in-depth analysis of such events in serum and other biofluids holds great promise for the discovery of new phosphoprotein and endogenous phosphopeptide biomarkers for diseases such as PDAC.

## Data Availability

The MS raw data files for this work were uploaded to ProteomeXchange (Identifier ID: PXD046720 and PXD045146).

## Supporting Information Available

Details of materials and methods; full ATR spectra ofthe MCM-48 beads and particle size distribution of Zr-IMAC@MCM-48 beads (Figure S1); MALDI-TOFMS spectra for digests of β-casein and/or BSA without or with enrichment (FigureS2); comparisonof Zr-IMAC@MCM-48 with Ti-IMAC@MCM-48 and the commercial Fe-NTA and TiO_2_ beads (Figure S3); SDS-PAGEanalysis of samples and performance evaluation of the RPLC column (Figure S4); representativeMS^2^ spectra of endogenous phosphopeptides (Figure S5); reproducibility evaluation of Zr-IMAC@MCM-48 material (Figure S6); dynamicrange of the identified endogenous phosphopeptides in serum (Figure S7); phosphopeptidesidentified from the tryptic digest of α-casein and β-casein and its mixturespiked in human serum enriched by Zr-IMAC@MCM-48 (Table S1); comparisonof endogenous phosphopeptides identified from serum with previous reports (TableS2) (PDF)
Identification of endogenous phosphopeptides and phosphosites (Table S3) (XLSX)
Quantification of endogenous phosphopeptides from serum of PDAC and control groups (Table S4) (XLSX)
Quantification of trypsin digestion-derived phosphopeptides from serum of PDAC and controlgroups (Table S5) (XLSX)
