# Supplementary material for: In-Depth Endogenous Phosphopeptidomics of Serum with Zirconium(IV)-Grafted Mesoporous Silica Enrichment
Source: Anal Chem. 2024 May 10;96(21):8254–62. doi: 10.1021/acs.analchem.3c02150 (PMC11140682; doi:10.1021/acs.analchem.3c02150)
Supplement: Supplementary file 1 — ac3c02150_si_001.pdf [file ac3c02150_si_001.pdf]

Supporting Information

In-depth Endogenous Phosphopeptidomics of Serum with Zirconium (IV)-Grafted Mesoporous Silica Enrichment

Ci Wu, [+, ‡] Shen Zhang,[§, ‡] Chunyan Hou, [+] Stephen Byers, [+] Junfeng Ma, [+*]

[+] Department of Oncology, Lombardi Comprehensive Cancer Center, Georgetown University Medical Center, Washington DC 20007, USA.

[§] Clinical Research Center for Reproduction and Genetics in Hunan Province, Reproductive and Genetic Hospital of CITIC-XIANGYA, Changsha, 410000, China

[*] To whom correspondence should be addressed: Tel: +1-202-6873802; e-mail: junfeng.ma@georgetown.edu

[‡] These authors contributed equally.

Table of Content

**Description**	**Page No.**
Experimental section	S-3
Figure S1. Full ATR spectra (4500-750 cm^{-1}) of the MCM-48 beads (A) and particle size distribution of Zr-IMAC@MCM-48 beads (B).	S-8
Figure S2. MALDI-TOF MS spectra for digests of β-casein and/or BSA without or with enrichment	S-9
Figure S3. Comparison of Zr-IMAC@MCM-48 with Ti-IMAC@MCM-48 and the commercial Fe-NTA and TiO_2 beads for the enrichment of phosphopeptides from HeLa cell digests.	S-10
Figure S4. (A) SDS-PAGE analysis of samples after enrichment of endogenous phosphopeptides from serum; (B) The performance evaluation of the RPLC column before, during, and after analysis of 10 serum endogenous phosphopeptide samples.	S-11
Figure S5. Representative MS^2 spectra of endogenous phosphopeptides.	S-12
Figure S6. Reproducibility evaluation of Zr-IMAC@MCM-48 material for the enrichment of phosphopeptides from the digest of HeLa cell lysates and endogenous phosphopeptides from serum.	S-13
Figure S7. Dynamic range of the identified endogenous phosphopeptides in serum.	S-14
Table S1. Phosphopeptides identified from the tryptic digest of α-casein & β-casein and its mixture spiked in human serum enriched by Zr-IMAC@MCM-48.	S-15
Table S2. Comparison of endogenous phosphopeptides identified from serum with previous reports.	S-17
References	S-19
Table S3. Identification of endogenous phosphopeptides and phosphosites.	
Table S4. Quantification of endogenous phosphopeptides from serum of PDAC and control groups.	
Table S5. Quantification of trypsin digestion-derived phosphopeptides from serum of PDAC and control groups.	

EXPERIMENTAL SECTION

Chemicals and Materials

3-aminopropyltriethoxysilane (APTES, 97%), (aminomethyl) phosphonic acid (AMPA, 99%), bovine serum album (BSA), α-casein and β-casein (bovine milk), DTT (1,4-dithiothreitol), pyrrolidine, ammonium hydroxide solution (v/v, ~28%), pepstatin A, bestatin, marimastat, mesoporous silica MCM-48 (15 μm of particle size, 3 nm of pore size) were purchased from Sigma (St. Louis, MO). Methane sulfonic acid (98%), sodium cyanoborohydride ($NaCNBH_3$), Zirconium (IV) sulfate $Zr(SO_4)_2$, Titanium (IV) sulfate $Ti(SO_4)_2$, and $FeCl_3$ were obtained from Alfa Aesar. Iodoacetamide (IAA) and glutaraldehyde (GA, 50% solution) were ordered from VWR. Titansphere Phos-Tio Kit (TiO_2) was obtained from GL Sciences (Tokyo, Japan). Ni-NTA silica resin was purchased from (QIAGEN). ATR measurements of the beads were performed with a 6700 FT-IR spectrometer (Thermo Fisher Scientific). Pore diameter and specific surface area were characterized through nitrogen adsorption-desorption isotherms utilizing a QuadraSorb SI4 instrument (FL, USA). Particle size distribution of Zr-IMAC@MCM-48 beads was measured on BT-9300ST Laser Particle Size Analyzer (Dandong Bettersize Instruments Ltd., China). Pooled human serum was purchased from Innovative Research, Inc (Novi, MI). Five PDAC and five matched-control serum samples were obtained from the Tissue Culture & Bio-banking Shared Resource, Georgetown University Medical Center.

Preparation of Zirconium (IV)-Immobilized MCM-48

The synthetic procedure for zirconium (IV)-immobilized MCM-48 material was referred to our previous work,[1] with some modifications (Figure 1a). In brief, the MCM-48 (1 g) was first activated by refluxing in 33% (v/v) methanesulfonic acid for 24 h, then washed thoroughly with deionized H_2O and dried at 70 °C overnight. The activated MCM-48 material was refluxed with 2 mL of APTES and 40 mL of dry toluene for 16 h under continuous stirring and heating (110 °C). The material was then washed with ethanol followed by drying at 70 °C overnight. The resulting APTES@MCM-48 was stirred with 10 mL of 10% glutaraldehyde in 100 mM PBS (pH 8.0) for 6 h at 25 °C followed by

washing with deionized water. The glutaraldehyde activated material was then stirred Finally, zirconium (IV) was immobilized by incubation in 10 mL 100 mM $Zr(SO_4)_2$ at 25 °C for 6 h. The resulting material was then washed with H_2O and dried at 70 °C overnight. Additionally, the Ti-IMAC@MCM-48 and Fe-IMAC@MCM-48 were synthesized by incubating with $Ti(SO_4)_2$ and $FeCl_3$, respectively. As another comparison, Fe-NTA was synthesized by derivatization with Ni-NTA agarose beads (Qiagen) according to a previous report.[2] The Ni-NTA agarose beads were first de-chelated in 100 mM EDTA by incubating in room temperature for 30 min, followed by washing with water to remove the EDTA. Then the material was chelated by incubating with 10 mM $FeCl_3$ for 30 min.

Enrichment of phosphopeptides from standard proteins with Zr-IMAC@MCM-48

The β-casein digest (2 μg) and the mixture interfered with BSA digests at mass ratio 1:1000) were used to evaluate enrichment selectivity. Two mg of material was first conditioned with 200 μL of the loading buffer consisting of 80% ACN, 5% TFA, and 0.1 M glycolic acid. The sample was dissolved in 200 μL loading buffer loaded onto the beads, and incubated for 45 min. The suspension was centrifuged at 5,000 rpm for 2 min, with beads washed with 400 μL of 80% acetonitrile and 1% TFA followed by 400 μL of 0.1% TFA. Enriched phosphopeptides were eluted with 20 μL of 1% ammonium hydroxide and 20 μL of 5% pyrrolidine. 2 μL of the combined eluates were used for matrix assisted laser desorption ionization-time of flight-mass spectrometry (MALDI TOFMS) analysis with DHB matrix.

Digests of standard protein mixture (α-casein & β-casein, 10 μg) with or without being spiked in 20 μL serum were also subjected to enrichment using the above method. The obtained eluates were desalted and lyophilized to dryness prior to nanoRPLC-MS/MS analysis.

Enrichment of phosphopeptides from HeLa cell digests with TiO_2 and Fe-NTA

IMAC materials were first conditioned with 200 μL of the loading buffer consisting of 80% ACN, 5% TFA, and 0.1 M glycolic acid. Two mg of Zr-IMAC@MCM-48, Ti-

IMAC@MCM-48, and Fe-IMAC@MCM-48 were used. Digests of HeLa cell lysates (100 μg), and serum were dissolved in 200 μL loading buffer, loaded onto the beads, and incubated for 45 min. The suspension was centrifuged at 5,000 rpm for 2 min, with beads washed with 400 μL of 80% acetonitrile and 1% TFA followed by 400 μL of 0.1% TFA. Enriched phosphopeptides were eluted with 50 μL of 1% ammonium hydroxide and 50 μL of 5% pyrrolidine. The combined eluates were desalted and lyophilized to dryness.

As a comparison, enrichment with TiO_2 spin tip (200 μL) was performed as well, by following the manufacturer's manual. The Fe-NTA enrichment was performed according to the conditions described by Chen et al.[2–4] with minor modifications. Briefly, 20 mg of Fe-NTA was first equilibrated with a loading buffer containing 6% acetic acid and incubated with the sample dissolved in loading buffer for 30 minutes at 25 °C. The sample-beads mixture was then loaded onto a 200 μL tip containing 20 μm polypropylene frits and subjected to centrifugation (3300 g for 3 minutes at room temperature). Following successive washes with 100 μL of washing buffer (loading buffer: ACN = 3:1) and 0.5% acetic acid, the bound peptides were eluted twice from the IMAC tip using 100 μL of 200 mM $NH_4H_2PO_4$. The eluted peptides were then desalted using reversed-phase StageTips, and dried under vacuum.

MALDI-TOF Analysis

Analysis of the simple protein β-casein digest with or without interference of BSA digest was conducted by matrix-assisted laser desorption/ionization-time of flight (MALDI-TOF) MS on an Ultraflex III TOF/TOF mass spectrometer (Bruker Daltonics; Bremen, Germany). A matrix solution containing 2,5-dihydroxybenzoic acid (DHB, 25 mg/mL) was prepared in $ACN/H_2O/H_3PO_4$ (60:40:1, v/v/v). Equal volumes (2 μL) of the sample and DHB were sequentially deposited onto the MALDI plate for mass-spectrometric analysis. Spectra were acquired in positive-ionization mode using reflector detection.

NanoRPLC-MS/MS and Data Analysis

For MS analyses of phosphopeptides from α-casein & β-casein with or without serum interference and HeLa cell lysates, a Thermo Fisher Orbitrap Eclipse Tribrid mass spectrometer (MS) equipped with a FAIMS Pro Interface was used to conduct experiments. Digested peptides were directly loaded on a 2 cm PEPMAP trap column (ThermoFisher Scientific) for online desalting. Besides, separation was performed on a 25 cm PepMap analytical column (ThermoFisher Scientific) with 3% to 38% buffer B (80% ACN, 0.1% formic acid) for 102 min and kept at 100% buffer B for 10min. FAIMS switched between CVs of −35 V, -45V and −65 V with 1 s cycle time. MS1 spectra were obtained in the Orbitrap (resolution: 60k; AGQ target: standard; MaxIT: Auto; RF lens: 50%; mass range: 350 to 1,500). Dynamic exclusion was used for 40 s to exclude all charge states for a specified precursor. The collection of MS2 spectra was completed in the linear ion trap (isolation window: 1.6 m/z; scan rate: rapid; AGQ target: standard; MaxIT: Auto; HCD CE: 35%; data type: centroid).

While phosphopeptides derived from tryptic digestion of serum proteins and endogenous phosphopeptides directly extracted from serum samples were analyzed with a system coupling the nanoAcquity UPLC (Waters) and the Orbitrap Fusion Lumos mass spectrometer (Thermo Fisher), as described previously.[5] In brief, a 150-min gradient of mobile phase A (0.1% formic acid in 2% ACN) and mobile phase B (0.1% formic acid in ACN) was used for separation. All the MS data were acquired in data dependent acquisition mode. Database searching of the raw files was performed in Proteome Discoverer 2.5 (Thermo Fisher Scientific) with the Sequest HT search engine by using the database of Homo sapiens (TaxID 9606, Release 2017-07-05, 42253 sequences, Uniprot). The database-searching parameters of phosphopeptides from HeLa cells lysates were set as below: full tryptic digestion and allowed up to two missed cleavages, the precursor mass tolerance was set at 10 ppm, whereas the fragment-mass tolerance was set at 0.02 Da. Carbamidomethylation of cysteines (+57.0215 Da) was set as a fixed modification, and variable modifications of methionine oxidation (+15.9949 Da), acetyl (N-terminus, +42.011 Da), and phosphorylation (serine (S), threonine (T) or tyrosine (Y), +79.966 Da) were allowed. The false-discovery rate (FDR) was determined by using a target-decoy search strategy. The decoy-sequence

database contains each sequence in reverse orientations, enabling FDR estimation. On the peptide level, the corresponding FDR was less than 1%. The phosphosites with greater than 0.75 localization probabilities were considered as confident identification.

The database-searching parameters of endogenous phosphopeptides were set as below: non-specific enzyme, the precursor mass tolerance was set at 10 ppm, whereas the fragment-mass tolerance was set at 0.02 Da. Variable modifications of methionine oxidation (+15.9949 Da), acetyl (N-terminus, +42.011 Da), and phosphorylation (T, S or Y, +79.966 Da) were allowed. Moreover, an FDR of less than 1% was applied at the peptide level. And phosphosites with >0.75 localization probabilities were regarded as confident identification. Label-free quantification (LFQ) method was used for the quantifying the phosphopeptides, and only phosphopeptides quantified in both PDAC and control groups were used for the volcano and PCA plots. The R packages "tidyverse" [6] and "ggfortify" [7] were used for plotting. P-values were calculated for five replicate data by using biological replicate as study factors in Proteome Discoverer 2.4, with -log 10 (p value) calculated and used as the y-axis value in the volcano plots. The dynamic range distribution was calculated using the log 10 intensity of the quantified endogenous phosphopeptides.

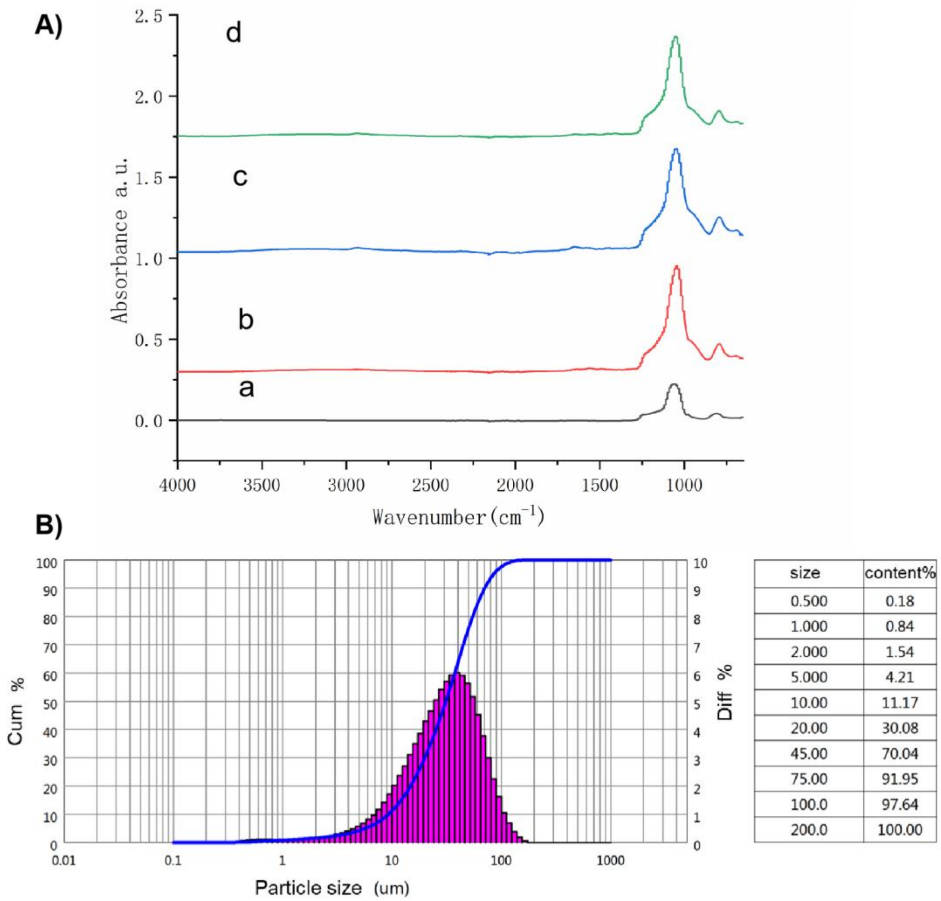

size	content%
0.500	0.18
1.000	0.84
2.000	1.54
5.000	4.21
10.00	11.17
20.00	30.08
45.00	70.04
75.00	91.95
100.0	97.64
200.0	100.00

Figure S1. (A) Full ATR spectra (4500-750 cm^{-1}) of the MCM-48 beads: (a) original MCM-48 beads, (b) APTES modified MCM-48 beads, (c) glutaraldehyde modified MCM-48 beads, and (d) AMPA modified MCM-48 beads. (B) Particle size distribution of Zr-IMAC@MCM-48 beads (cum %, cumulative distribution; diff %, differential distribution).

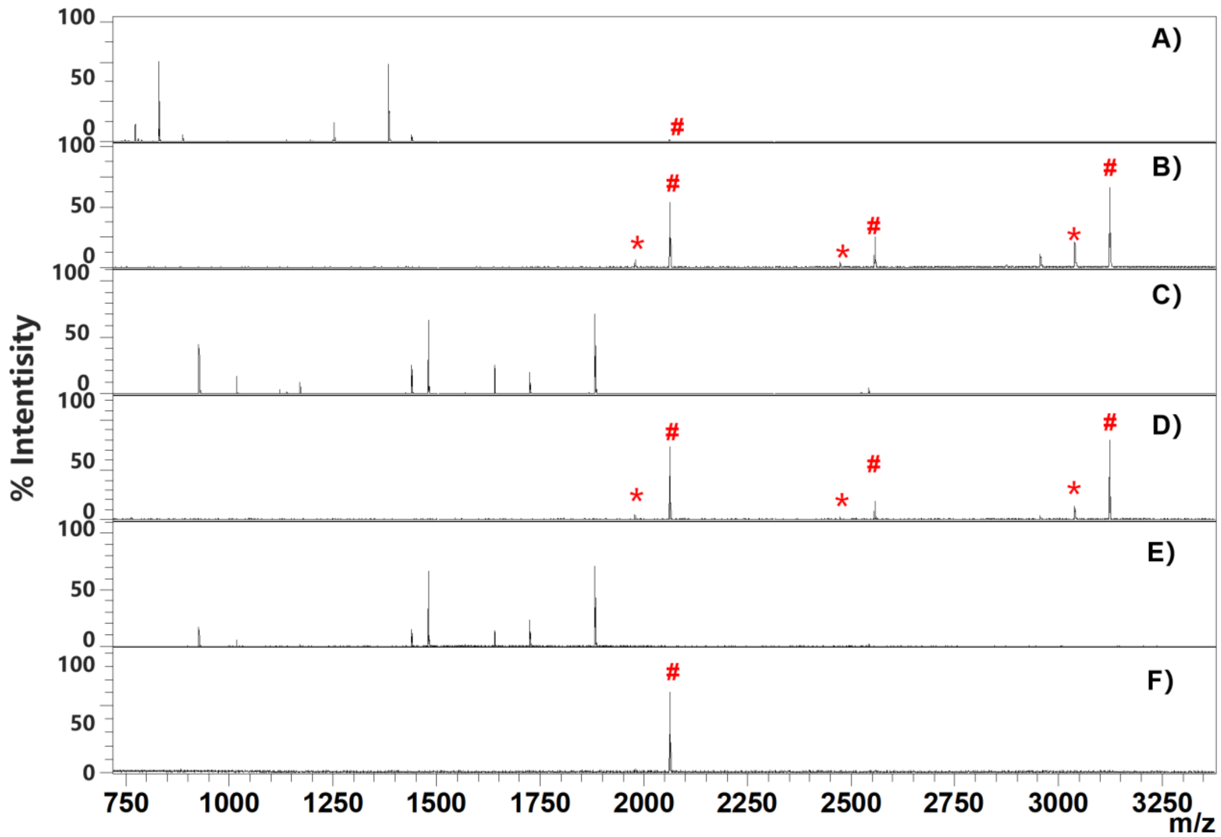

Figure S2. MALDI-TOF mass spectra: (A) direct analysis of β-casein tryptic digest, and (B) β-casein tryptic digest after enrichment by Zr-IMAC@MCM-48; (C) direct analysis of the mixture of BSA and β-casein digests at 1000:1 ratio, and (D) mixture of BSA and β-casein digests (1000:1) after enrichment by Zr-IMAC@MCM-48; (E) direct analysis of the mixture of BSA and β-casein digests at 5000:1 ratio, and (F) mixture of BSA and β-casein digests (5000:1) after enrichment by Zr-IMAC@MCM-48. Note: phosphopeptides are marked with red "#", while neutral loss peaks are marked with red "*".

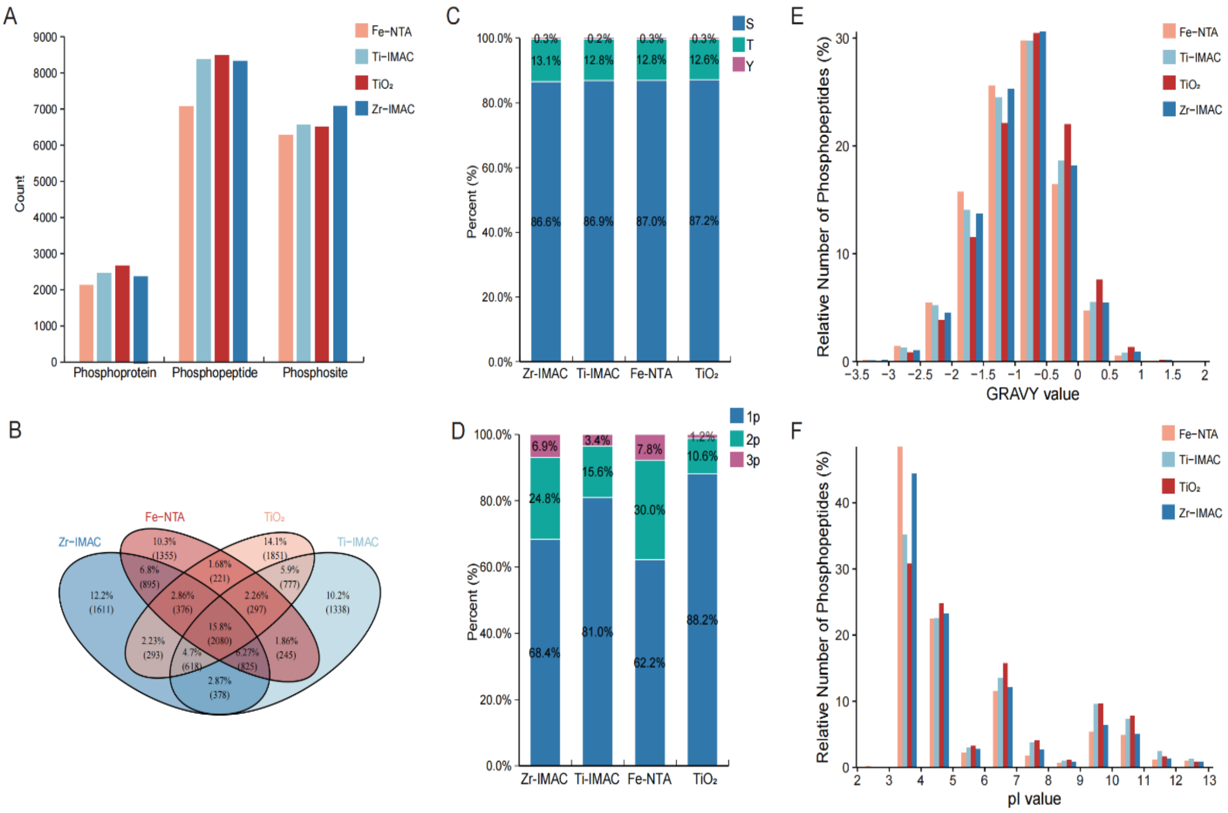

Figure S3. Comparison of Zr-IMAC@MCM-48 with Ti-IMAC@MCM-48, commercial Fe-NTA and TiO_2 beads for the enrichment of phosphopeptides from the digest of HeLa cell lysates. (A) Number of identified phosphopeptides, phosphoproteins and phophosites. (B) Venn diagram of the phosphosites identified by each method (the number of p-pep and percentage relative to total). (C) Distribution of singly- (1-P), doubly- (2-P) and triply- (3-P) phosphopeptides. (D) Distribution of phosphorylation on serine (S), threonine (T), and tyrosine (Y). (E)&(F) Distribution of the GRAVY and pI value of the phosphopeptides isolated with each method, respectively.

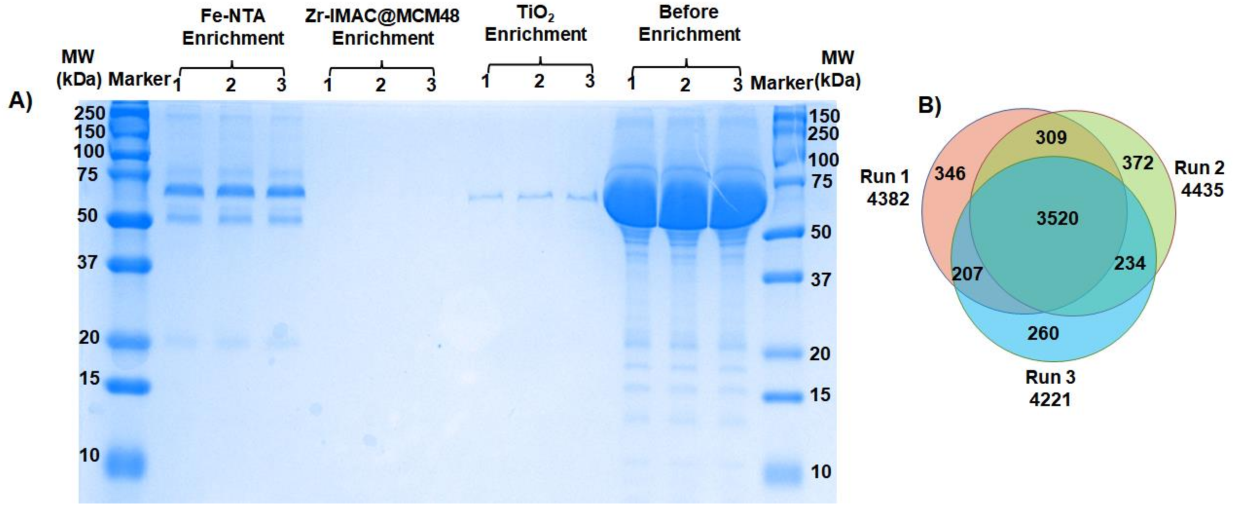

Figure S4. (A) SDS-PAGE analysis of samples after enrichment of endogenous phosphopeptides from serum by Fe-NTA, Zr-IMAC@MCM48, and TiO_2, respectively. Samples without enrichment were used as a control. (B) The performance evaluation of the HPLC column before, during, and after analysis of 10 endogenous phosphopeptide samples after Zr-IMAC@MCM48 enrichment from serum (QC sample: 200 ng standard HeLa cell digests obtained from Thermo Fisher Scientific).

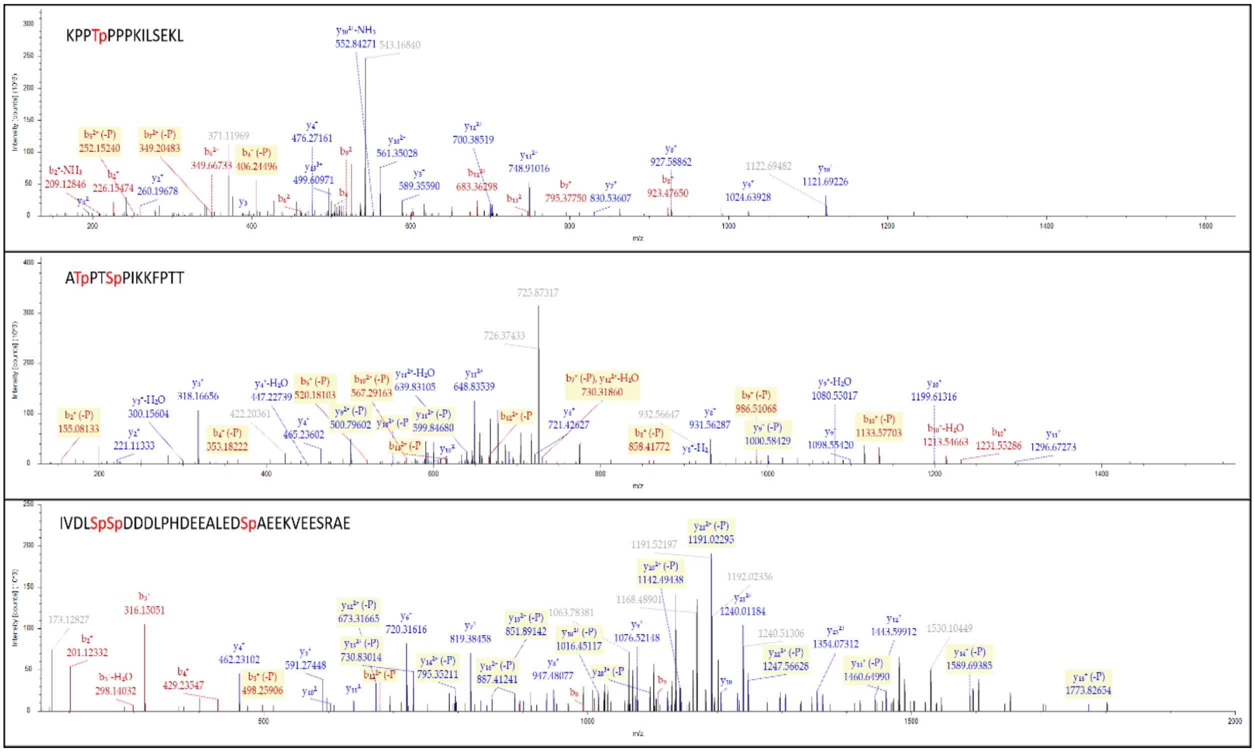

Figure S5. Representative MS^2 spectra of three endogenous phosphopeptides with one site, two sites, or three sites identified from human serum. The b- and y- ion series shown are indicated with red and blue marks to verify the identification of individual peptides.

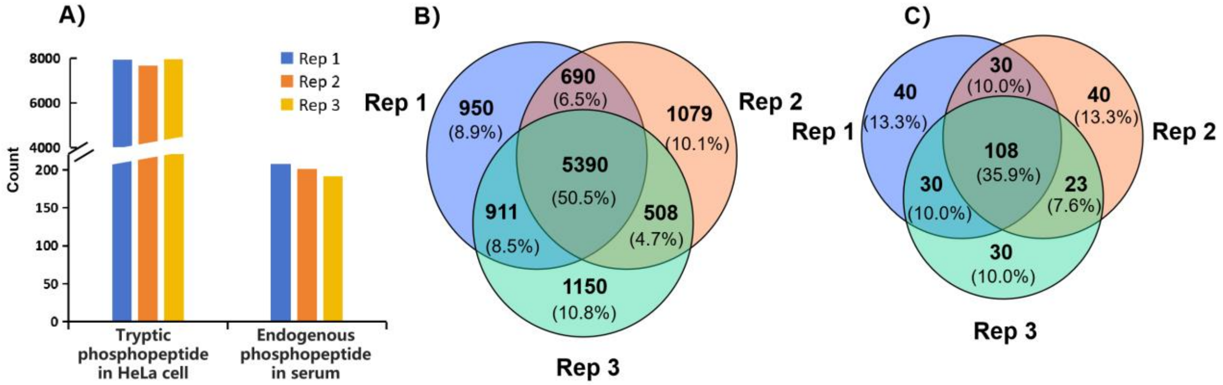

Figure S6. Reproducibility evaluation of Zr-IMAC@MCM-48 material for the enrichment of phosphopeptides from the tryptic digest of HeLa cell lysates and endogenous phosphopeptides from serum. (A) Number of identified phosphopeptides. (B) Venn diagram of the phosphopeptides identified from the digest of HeLa cell lysates across three replicates. (C) Venn diagram of the endogenous phosphopeptides identified from serum across three replicates.

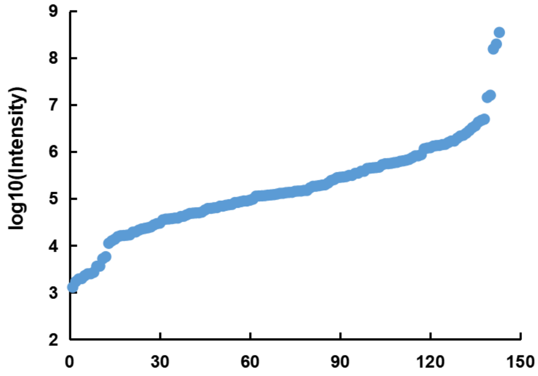

Figure S7. Dynamic range of the identified endogenous phosphopeptides in serum.

Table S1. Phosphopeptides identified from the tryptic digest of α-casein & β-casein and its mixture spiked in human serum enriched by Zr-IMAC@MCM-48.

No.	Amino Acid Sequence	Peptide m/z	No. of phospho -sites	Standard protein digest	Spiked in serum
α1	EKVNELSpKDIGSpESpTEDQAMEDIK	2935.16	3	Y	Y
α2	VNELSpKDIGSpESpTEDQAMEDIK	2678.023	3	Y	Y
α3	EQLSpTpSEENSKKTVDMESpTEVFTK	2987.192	3	Y	
α4	NAVPITPTLNREQLSTpSpEENSK	2588.164	2	Y	Y
α5	DIGSpESpTEDQAMEDIK	1927.692	2	Y	Y
α6	NAVPITPTpLNREQLSpTSEENSKK	2716.259	2	Y	Y
α7	EKVNELSKDIGSpESpTEDQAMEDIK	2855.194	2	Y	Y
α8	VNELSKDIGSpESpTEDQAMEDIK	2598.057	2	Y	
α9	NAVPITPTLNREQLSpTSEENSKK	2636.293	1	Y	Y
α10	KTVDMESpTEVFTKK	1722.802	1	Y	Y
α11	TVDMESpTEVFTK	1466.612	1	Y	Y
α12	TVDMESpTEVFTKK	1594.707	1	Y	Y
α13	DIGSESpTEDQAMEDIK	1847.725	1	Y	Y
α14	KYKVPQLEIVPNSpAEER	2080.047	1	Y	Y
α15	YKVPQLEIVPNSpAEER	1951.952	1	Y	Y
α16	YKVPQLEIVPNSpAEERLHSMK	2548.263	1	Y	Y
α17	VPQLEIVPNSpAEER	1660.794	1	Y	Y
α18	VPQLEIVPNSpAEERLHSMK	2257.105	1	Y	
α19	KTVDMoESpTEVFTKK	1738.797	1	Y	Y

α20	TVDMoESpTEVFTKK	1610.702	1	Y	Y
α21	TVDMoESpTEVFTK	1482.607	1	Y	Y
α22	EKVNELSpKDIGSpESpTEDQAMoEDIK	2951.155	3	Y	
α23	YKVPQLEIVPNSpAEERLHSMoK	2564.258	1	Y	Y
α24	VNELSKDIGSpESpTEDQAMoEDIK	2614.051	2	Y	Y
α25	VPQLEIVPNSpAEERLHSMoK	2273.1	1	Y	
α26	DIGSpESpTEDQAMoEDIK	1943.686	2	Y	Y
α27	DIGSpESTEDQAMoEDIK	1863.72	1	Y	
β1	KIEKFQSpEEQQQTEDELQDK	2560.145	1	Y	Y
β2	RELEELNVPGEIVESpLSpSpSpEESITR	3122.27	3	Y	Y
β3	FQSpEEQQQTEDELQDK	2061.828	1	Y	Y

Note: S or T with p, phosphorylated site; Mo, oxidation on methionine.

Table S2. Comparison of endogenous phosphopeptides identified from serum with previous reports.

No.	Enrichment material	No. of Endogenous Phosphopeptides	Enrichment Selectivity	Ref.
1	Titanium (IV)-Immobilized Mesoporous Silica Particles (MCM41)	4	-	[8]
2	Ti^{4+}-EPO nanoparticles	4	-	[9]
3	Ti^{4+}-immobilized Fe_3O_4@polydopamine core–shell microspheres	6	-	[10]
4	Combining size-selective Ti (IV)-MCM-41 enrichment, high-pH RP–RP off-line separation, and complementary CID and ETD fragmentation	143	20.2%	[11]
5	Titanium grafted magnetic mesoporous silica (Fe_3O_4@Ti-mSiO_2)	4	-	[12]
6	TiO_2 nanotube array-integrated portable microdevice	4	-	[13]
7	SiO_2–TiO_2 composite fiber	4	-	[14]
8	Yolk–shell Fe_3O_4@mTiO_2@mSiO_2 nanocomposite	4	-	[15]
9	Magnetic graphene/mesoporous silica composites with titanium(IV)-immobilized pore walls (denoted as Ti^{4+}-MGMSs)	4	-	[16]
10	Fe_3O_4@PDA@Zr-MOF composites	4	-	[17]
11	Titanium(IV)-Immobilized Hydrophilic Hierarchically Ordered Macro-/Mesoporous Silica	4	-	[18]
12	Carboxyl cotton chelator-titanium(IV) (CCC-Ti^{4+}) fibers	4	-	[19]

13	Hydrophilic magnetic graphene@metal–organic framework (MOF)	4	-	[20]
14	Zr-based metal–organic frameworks (MOFs) of UiO-66	4	-	[21]
15	Core–Shell Magnetic Metal–Organic Framework Nanoparticles (Fe_3O_4@MIL-100 (Fe))	4	-	[22]
16	Diamond-lanthanide metal oxide affinity composites	4	-	[23]
17	Mesocrystalline SnO_2 Nanorods on Reduced Graphene Oxide Sheets	4	-	[24]
18	Adenosine phosphate-Ti^{4+} functionalized magnetic mesoporous graphene oxide nanocomposite (denoted as MG@mSiO2-ATP-Ti^{4+})	4	-	[25]
19	Dendritic mesoporous silica nanoparticles modified with polydopamine and chelated Ti^{4+} (denoted DMSNs@PDATi^{4+})	4	-	[26]
20	Direct dilution protocol and the Ti^{4+}-IMAC magnetic material enrichment	176	23%	[27]
21	Ti-based MOF nanosheet	4	-	[28]
22	Paper-based Phos-PAD	4	-	[29]
23	Zr-IMAC@MCM-48	329	61%	This work

REFERENCES:

(1) Hou, C.; Ma, J.; Tao, D.; Shan, Y.; Liang, Z.; Zhang, L.; Zhang, Y. *J. Proteome Res.* **2010**, *9* (8), 4093–4101.

(2) Tsai, C.-F.; Hsu, C.-C.; Hung, J.-N.; Wang, Y.-T.; Choong, W.-K.; Zeng, M.-Y.; Lin, P.-Y.; Hong, R.-W.; Sung, T.-Y.; Chen, Y.-J. *Anal. Chem.* **2014**, *86* (1), 685–693.

(3) Tsai, C.-F.; Wang, Y.-T.; Yen, H.-Y.; Tsou, C.-C.; Ku, W.-C.; Lin, P.-Y.; Chen, H.-Y.; Nesvizhskii, A. I.; Ishihama, Y.; Chen, Y.-J. *Nat. Commun.* **2015**, *6* (1), 6622.

(4) Kitata, R. B.; Choong, W.-K.; Tsai, C.-F.; Lin, P.-Y.; Chen, B.-S.; Chang, Y.-C.; Nesvizhskii, A. I.; Sung, T.-Y.; Chen, Y.-J. *Nat. Commun.* **2021**, *12* (1), 2539.

(5) Wu, C.; Shi, S.; Hou, C.; Luo, Y.; Byers, S.; Ma, J. *ACS Appl. Mater. Interfaces* **2022**, *14* (42), 47482–47490.

(6) Wickham, H.; Averick, M.; Bryan, J.; Chang, W.; McGowan, L.; François, R.; Grolemund, G.; Hayes, A.; Henry, L.; Hester, J.; Kuhn, M.; Pedersen, T.; Miller, E.; Bache, S.; Müller, K.; Ooms, J.; Robinson, D.; Seidel, D.; Spinu, V.; Takahashi, K.; Vaughan, D.; Wilke, C.; Woo, K.; Yutani, H. *JOSS* **2019**, *4* (43), 1686.

(7) Yuan, T.; Masaaki, H.; Wenxuan, L. *The R Journal 8.2* **2016**, *8(2)*, 478–489.

(8) Hu, L.; Zhou, H.; Li, Y.; Sun, S.; Guo, L.; Ye, M.; Tian, X.; Gu, J.; Yang, S.; Zou, H. *Anal. Chem.* **2009**, 81 (1), 94–104.

(9) Qin, H.; Wang, F.; Wang, P.; Zhao, L.; Zhu, J.; Yang, Q.; Wu, R.; Ye, M.; Zou, H. *Chem. Commun.* **2012**, 48 (7), 961–963.

(10) Yan, Y.; Zheng, Z.; Deng, C.; Zhang, X.; Yang, P. *Chem. Commun.* **2013**, 49 (44), 5055.

(11) Zhu, J.; Wang, F.; Cheng, K.; Song, C.; Qin, H.; Hu, L.; Figeys, D.; Ye, M.; Zou, H. *Proteomics* **2013**, 78, 389–397.

(12) Li, X.-S.; Pan, Y.-N.; Zhao, Y.; Yuan, B.-F.; Guo, L.; Feng, Y.-Q. *J. Chromatogr. A* **2013**, 1315, 61–69.

(13) Min, Q.; Chen, X.; Zhang, X.; Zhu, J.-J. *Lab Chip* **2013**, 13 (19), 3853.

(14) He, X.-M.; Zhu, G.-T.; Li, X.-S.; Yuan, B.-F.; Shi, Z.-G.; Feng, Y.-Q. *Analyst* **2013**, 138 (18), 5495.

(15) Wan, H.; Li, J.; Yu, W.; Liu, Z.; Zhang, Q.; Zhang, W.; Zou, H. *RSC Adv.* **2014**, 4 (86), 45804–45808.

(16) Sun, N.; Deng, C.; Li, Y.; Zhang, X. *ACS Appl. Mater. Interfaces* **2014**, 6 (14), 11799–11804.

(17) Zhao, M.; Deng, C.; Zhang, X. *Chem. Commun.* **2014**, 50 (47), 6228.

(18) Yan, Y.; Zheng, Z.; Li, Y.; Deng, C.; Zhang, X. *ChemPlusChem* **2014**, 79 (5), 662–666.

(19) He, X.-M.; Chen, X.; Zhu, G.-T.; Wang, Q.; Yuan, B.-F.; Feng, Y.-Q. *ACS Appl. Mater. Interfaces* **2015**, 7 (31), 17356–17362.

(20) Zhao, M.; Zhang, X.; Deng, C. *RSC Adv.* **2015**, 5 (45), 35361–35364.

(21) Zhu, X.; Gu, J.; Yang, J.; Wang, Z.; Li, Y.; Zhao, L.; Zhao, W.; Shi, J. *J. Mater. Chem. B* **2015**, 3 (20), 4242–4248.

(22) Chen, Y.; Xiong, Z.; Peng, L.; Gan, Y.; Zhao, Y.; Shen, J.; Qian, J.; Zhang, L.; Zhang, W. *ACS Appl. Mater. Interfaces* **2015**, 7 (30), 16338–16347.

(23) Hussain, D.; Musharraf, S. G.; Najam-ul-Haq, M. *Anal Bioanal Chem* **2016**, 408 (6), 1633–1641.

(24) Ma, W.; Zhang, F.; Li, L.; Chen, S.; Qi, L.; Liu, H.; Bai, Y. *ACS Appl. Mater. Interfaces* **2016**, 8 (51), 35099–35105.

(25) Su, J.; He, X.; Chen, L.; Zhang, Y. *ACS Sustainable Chem. Eng.* **2018**, 6 (2), 2188–2196.

(26) Hong, Y.; Yao, Y.; Zhao, H.; Sheng, Q.; Ye, M.; Yu, C.; Lan, M. *Anal. Chem.* **2018**, 90 (12), 7617–7625.

(27) La Barbera, G.; Capriotti, A. L.; Cavaliere, C.; Ferraris, F.; Laus, M.; Piovesana, S.; Sparnacci, K.; Laganà, A. *Anal. Bioanal. Chem.* **2018**, 410 (3), 1177–1185.

(28) Gu, Z.-Y. *Anal. Chem.* **2018**, 90 (22), 13796–13805.

(29) Li, L.; Geng, Y.; Xiang, Y.; Qiang, H.; Wang, Y.; Chang, J.; Zhao, H.; Zhang, L. *Anal. Chim. Acta* **2019**, 1062, 102–109.
